# Molecular classification reveals the sensitivity of lung adenocarcinoma to radiotherapy and immunotherapy: multi-omics clustering based on similarity network fusion

**DOI:** 10.1007/s00262-024-03657-x

**Published:** 2024-03-02

**Authors:** Jianguo Zhang, Yangyi Li, Weijing Dai, Fang Tang, Lanqing Wang, Zhiying Wang, Siqi Li, Qian Ji, Junhong Zhang, Zhengkai Liao, Jing Yu, Yu Xu, Jun Gong, Jing Hu, Jie Li, Xiuli Guo, Fajian He, Linzhi Han, Yan Gong, Wen Ouyang, Zhihao Wang, Conghua Xie

**Affiliations:** 1 Department of Pulmonary Oncology, Zhongnan Hospital of Wuhan University, Wuhan, 430071 Hubei China; 2 Department of Gastroenterology, Qingdao Municipal Hospital, Qingdao, 266000 Shandong China; 3 Tumor Precision Diagnosis and Treatment Technology and Translational Medicine, Hubei Engineering Research Center, Zhongnan Hospital of Wuhan University, Wuhan, 430071 Hubei China; 4 Human Genetics Resource Reservation Center, Wuhan University, Wuhan, 430071 Hubei China; 5 Hubei Key Laboratory of Tumour Biological Behaviors, Zhongnan Hospital of Wuhan University, Wuhan, 430071 Hubei China

**Keywords:** Lung adenocarcinoma, Multi-omics, Immunotherapy, Radiotherapy, Molecular subtype

## Abstract

**Background:**

Due to individual differences in tumors and immune systems, the response rate to immunotherapy is low in lung adenocarcinoma (LUAD) patients. Combinations with other therapeutic strategies improve the efficacy of immunotherapy in LUAD patients. Although radioimmunotherapy has been demonstrated to effectively suppress tumors, the underlying mechanisms still need to be investigated.

**Methods:**

Total RNA from LUAD cells was sequenced before and after radiotherapy to identify differentially expressed radiation-associated genes. The similarity network fusion (SNF) algorithm was applied for molecular classification based on radiation-related genes, immune-related genes, methylation data, and somatic mutation data. The changes in gene expression, prognosis, immune cell infiltration, radiosensitivity, chemosensitivity, and sensitivity to immunotherapy were assessed for each subtype.

**Results:**

We used the SNF algorithm and multi-omics data to divide TCGA-LUAD patients into three subtypes. Patients with the CS3 subtype had the best prognosis, while those with the CS1 and CS2 subtypes had poorer prognoses. Among the strains tested, CS2 exhibited the most elevated immune cell infiltration and expression of immune checkpoint genes, while CS1 exhibited the least. Patients in the CS2 subgroup were more likely to respond to PD-1 immunotherapy. The CS2 patients were most sensitive to docetaxel and cisplatin, while the CS1 patients were most sensitive to paclitaxel. Experimental validation of signature genes in the CS2 subtype showed that inhibiting the expression of RHCG and TRPA1 could enhance the sensitivity of lung cancer cells to radiation.

**Conclusions:**

In summary, this study identified a risk classifier based on multi-omics data that can guide treatment selection for LUAD patients.

**Supplementary Information:**

The online version contains supplementary material available at 10.1007/s00262-024-03657-x.

## Introduction

According to the global cancer data released by the International Agency for Research on Cancer and World Health Organization, there were 19.29 million new cancer cases and 9.96 million cancer-related deaths worldwide in 2020. The incidence rate of lung cancer was 11.4%, which was the second highest incidence rate of cancer worldwide, and the mortality rate was 18.0%, which was the highest mortality rate for cancer worldwide [1]. Although various treatments, including surgery, radiation therapy, chemotherapy, targeted therapy, and immunotherapy, have been substantially developed, the 5-year survival rate remains less than 18% [2], and the response rate to immunotherapy is still poor in patients with advanced lung cancer due to individual differences in tumors and immune systems [3]. Many preclinical and clinical studies have shown that radiotherapy can synergize with immunotherapy to boost its antitumor effects [4]. This combination significantly prolongs lung cancer patient progression-free survival and overall survival, indicating its broad prospects and considerable potential [5]. However, the underlying mechanisms are not fully understood. Increasing attention has been given to avoiding the immunosuppressive effects of radiotherapy to maximize its immunostimulatory impacts.

Classification of cancer subtypes according to molecular characteristics has become essential for understanding the heterogeneity of cancer and its impacts on diagnosis, prognosis, and treatment [6]. Traditional classification methods are primarily based on the anatomical site of origin. However, these approaches fail to capture the molecular variations that drive disease progression. Subtypes should be determined by not only the anatomical location but also specific genetic and protein changes within tumor cells. Molecular classification refers to grouping cancer based on its unique genetic, epigenetic, and proteomic characteristics [7]. Researchers have identified different subtypes with similar molecular characteristics and biological behaviors by studying the molecular features of tumors. These subtypes provide valuable insights into the potential mechanisms of cancer development and aid in the development of more targeted and personalized therapies. We have made notable advancements in our understanding of the biology of lung adenocarcinoma (LUAD) by categorizing various gene expression profiles at the transcriptional level [8]. The development of malignant transformation requires multilayered molecular changes, and single-level histological approaches are used to identify the mechanisms of cancer development through high-throughput techniques [9]. However, no single-molecule approach can fully explain the complexity of this problem [10]. Therefore, a multi-omics-based classification scheme for LUAD, which may uncover the heterogeneity of LUAD, has been proposed. This highlights the potential application of molecular classification in LUAD.

This study integrates transcriptomic, epigenetic, and somatic mutation data from LUAD patients to identify three subtypes using the similarity network fusion (SNF) algorithm and analyzes the differences between subgroups to characterize key events in LUAD development. Additionally, we discuss potential clinical treatment strategies based on specific molecular features, including chemotherapy and immunotherapy. This study provides a reference for precision medicine in LUAD patients.

## Materials and methods

### Cell culture and radiation

LUAD PC9 cells were purchased from the Type Culture Center of the Chinese Academy of Sciences (Shanghai, China), and A549 and H1299 cells were purchased from Procell (Wuhan, China). These cells were cultured in RPMI-1640 medium (HyClone Ltd., USA) supplemented with 10% fetal bovine serum. All cells were cultured in a standard tissue culture incubator at 37 °C with 95% humidity and 5% CO_2_. In the irradiation experiments, cells were exposed to a dose of 8 Gy using the Small Animal Radiation Research Platform (PXI X-RAD 225Cx, GulMay, CT, USA).

### RNA sequencing

A549 cells in a 10-cm cell culture dish, A549 cells were divided into two groups: the radiation group and the control group, with three replicates in each group. The radiation group received 8 Gy of X-ray irradiation. After 48 h, the samples were collected, and each sample was lysed with 1 mL of TrizolTRIzol (Vazyme, Nanjing, China) reagent to extract total RNA. Total RNA from six samples (three control and three radiation-treated samples) was sequenced on the BGISEQ platform (Beijing Genomics Institution, BGI). After filtering the reads with a PE150 sequencing length, HISAT was used to align the clean reads to the reference genome sequence. Bowtie2 was subsequently applied to align the clean reads to the reference gene sequence to obtain alignment results. Finally, differential expression analysis was performed using Deseq2.

### Data collection and processing

The RNA-seq TPM data of LUAD patients, including corresponding clinical data, were acquired from the TCGA and included 497 LUAD tissues and 54 normal tissues. Preprocessed methylation data were downloaded from the UCSC Xena database. Somatic mutation information was downloaded from the cBioPortal (https://www.cbioportal.org/). Copy number variation (CNV) data were collected from FireBrowse. After integrating the gene expression, methylation, mutation, and copy number variation data of 497 LUAD patients were integrated, the multi-omics dataset of 446 patients was ultimately selected for subsequent analysis. The NSCLC patient survival datasets (GSE31210, GSE68465, GSE37745, and GSE50081) were downloaded from the GEO database as the validation sets [11–14]. The GSE31210, GSE50081, and GSE37745 data were generated with the GPL570 platform (Affymetrix Human Genome U133 Plus 2.0 Array) using 226, 181, and 196 NSCLC tissue samples, respectively. The GSE68465 data were generated with the GPL96 platform (Affymetrix Human Genome U133A Array) using 442 LUAD samples. The immune-related genes were obtained from the MSigDB (immune system process and immune response).

### Identification of molecular subtypes

We conducted the data analysis following the official documentation of the R package "MOVICS" [15]. The molecular subtypes of LUAD were determined based on the expression of radiation-related genes and immune-related genes and on DNA methylation and somatic mutation data. Radiation-related genes were identified from among the genes differentially expressed in A549 cells after 8 Gy irradiation (Deseq2, logFC ≥ 1/logFC ≤ -1, Padj < 0.05). Differential analysis was performed on the DNA methylation CpG sites, followed by univariate Cox regression analysis to identify the CpG sites associated with overall survival (OS). The somatic mutation data included the 30 genes with the highest mutation frequencies. We analyzed the cluster prediction index (CPI) and gap statistic to determine the optimal number of cancer subtypes [16]. Next, we classified the multiple omics datasets using 10 clustering algorithms, namely, iClusterBayes, moCluster, CIMLR, IntNMF, ConsensusClustering, COCA, NEMO, PINSPlus, SNF, and LRA.

Similarity network fusion (SNF) is a novel computational method for data integration [17]. Working within the sample network space, SNF circumvents issues of differing scales, collection biases, and noise across various data types. The nonlinear integration of data enables SNFs to capitalize on the commonalities and complementary information present in different data types.

### Evaluation of genetic alterations among different subtypes

The CNV data of somatic cells were visualized using the "maftools" package in R [18]. The hg19.mat reference genome file was subsequently selected for annotation, and the GISTIC2.0 algorithm was used to assess the differences in genomic levels of loss and gain among different subtypes [19]. The tumor mutation burden (TMB) was obtained by calculating the number of nonsynonymous mutations per million bases. We used the built-in function of the MOVICS package to calculate the fraction of the genome altered (FGA) by copy number amplification or deletion [20].

### Immune cell infiltration and immune checkpoint analysis

The differences in immune cell infiltration among the three subtypes were evaluated using CIBERSORT and MCPcounte [21, 22]. Heatmaps revealed the differences in immune cell infiltration according to the different algorithms. In addition, the differences in immune checkpoint expression levels among these three subtypes were analyzed, further revealing the link between subtypes and immunity.

### Analysis of subtypes in relation to immunotherapy and chemotherapy sensitivity

We predicted the sensitivity of the samples to chemotherapy by using the Genomics of Drug Sensitivity in Cancer (GDSC) database. Using the R package pRRophetic, we estimated the sensitivity of LUAD patients to commonly used drugs, namely, cisplatin, paclitaxel, and docetaxel, through ridge regression [23]. The half-maximal inhibitory concentration (IC50) was used to compare the response to the drugs between subtypes. A specific gene set of 795 genes was obtained from a melanoma cohort of patients receiving CTLA-4 or PD-1 antibodies [24]. Subclass mapping analysis was also conducted to compare the similarities between the risk group and the immunotherapy subgroup, and patients who responded to anti-CTLA-4 or anti-PD-1 immunotherapy were identified [25].

### Analysis of single-cell RNA sequencing

The Seurat analysis pipeline provided by the R package scissor was used to analyze single-cell lung cancer data [26]. Cells with fewer than 200 genes and genes expressed in fewer than three cells were excluded, and the remaining cells were included for further analysis. Cell types were determined using marker genes from previous studies. Specifically, the tumor cell markers were EPCAM and KRT19; T/NK cell markers were NKG7, CD3E, CD3G, and CD3D; B-cell markers were CD79A and CD79B; myeloid cell marker was LYZ; mast cell markers were TPSB2 and TPSAB1; fibroblast markers were COL1A1 and COL1A2; endothelial cell marker was CLDN5; and normal epithelial cell marker was CAPS [27].

### The scissor algorithm combines single-cell data with bulk data

The scissor algorithm is used to identify cell subpopulations associated with a given phenotype from single-cell data [26]. We used the 'scissor' algorithm, we identified to identify scissor-positive and scissor-negative cells in single-cell data, which are related to the multi-omics molecular classification. These cells were denoted as '1' for scissor-positive and '2' for scissor-negative, while '0' represented cells unrelated to classification. Subsequently, we calculated the proportions of positive and negative cells across various cell subgroups. The functional differences between cell types were obtained by functional enrichment analysis of scissor + and scissor- cells. Then, using the R package cellChat, we calculated the cell communication intensity between scissor + and scissor- cells and the differences in receptor–ligand pairs [28].

#### External cohort validation

To validate the accuracy of our molecular classification predictions for prognosis, four LUAD datasets (GSE31210, GSE50081, GSE37745, and GSE68465) from the GEO were selected as external datasets for validation. The nearest template prediction (NTP) algorithm is flexibly applied to cross-platform, cross-species, and multicategory predictions without parameter optimization during analysis [29]. Therefore, the NTP algorithm was used to classify the external dataset molecularly and calculate the differences in prognosis among different classifications.

#### siRNA transfection

We transfected RHCG, TRPA1-specific, or TRPA1-nonspecific siRNAs synthesized by Beijing TsingKe Company (Beijing, China) using jetPRIME transfection reagent (Polyplus-transfection® SA, France). The siRNA sequences used are listed in Table S1.

#### RNA extraction and qRT‒PCR

Total RNA was isolated from cells using TRIzol reagent (Vazyme Ltd., China). We used HiScript® Q RT SuperMix (Vazyme Ltd., China) to transcribe RNA and ChamQTM SYBR® qPCR Master Mix (Vazyme Ltd., China) for qRT‒PCR. The primer sequences are listed in Table S2.

#### Flow cytometry

For apoptosis, the cells and medium supernatant were collected 48 h after treatment and washed twice with 4 °C PBS. The cells were stained with Annexin V-FITC and PI on ice. The cells were collected 24 h after treatment and washed with PBS for the cell cycle. The cells were then incubated with the staining agent PI at room temperature in the dark for 30 min. The data were acquired on the CytoFLEX system and analyzed with FlowJo V10.

#### Colony formation and cell counting kit-8 (CCK-8) assays

The cells were seeded into 6-well plates (1000 cells/well) and 96-well plates (1,000 cells/well) 48 h after radiation. A Cell Counting Kit-8 (CCK-8) assay was used to detect cell viability. After 10–14 days of culture, the colonies were fixed with 4% paraformaldehyde and stained with 0.5% crystal violet. The numbers of colonies were then counted via ImageJ.

#### Statistical analysis

All experimental data are expressed as the mean ± standard deviation (SD). One-way analysis of variance (ANOVA) was used to determine significant differences among more than two groups. Student’s t test was used to test for statistical significance between two groups, and *P* ≤ 0.05 was considered to indicate statistical significance.

## Results

### Three subtypes of LUAD patients were categorized by multi-omics classification

**Fig. 1:**
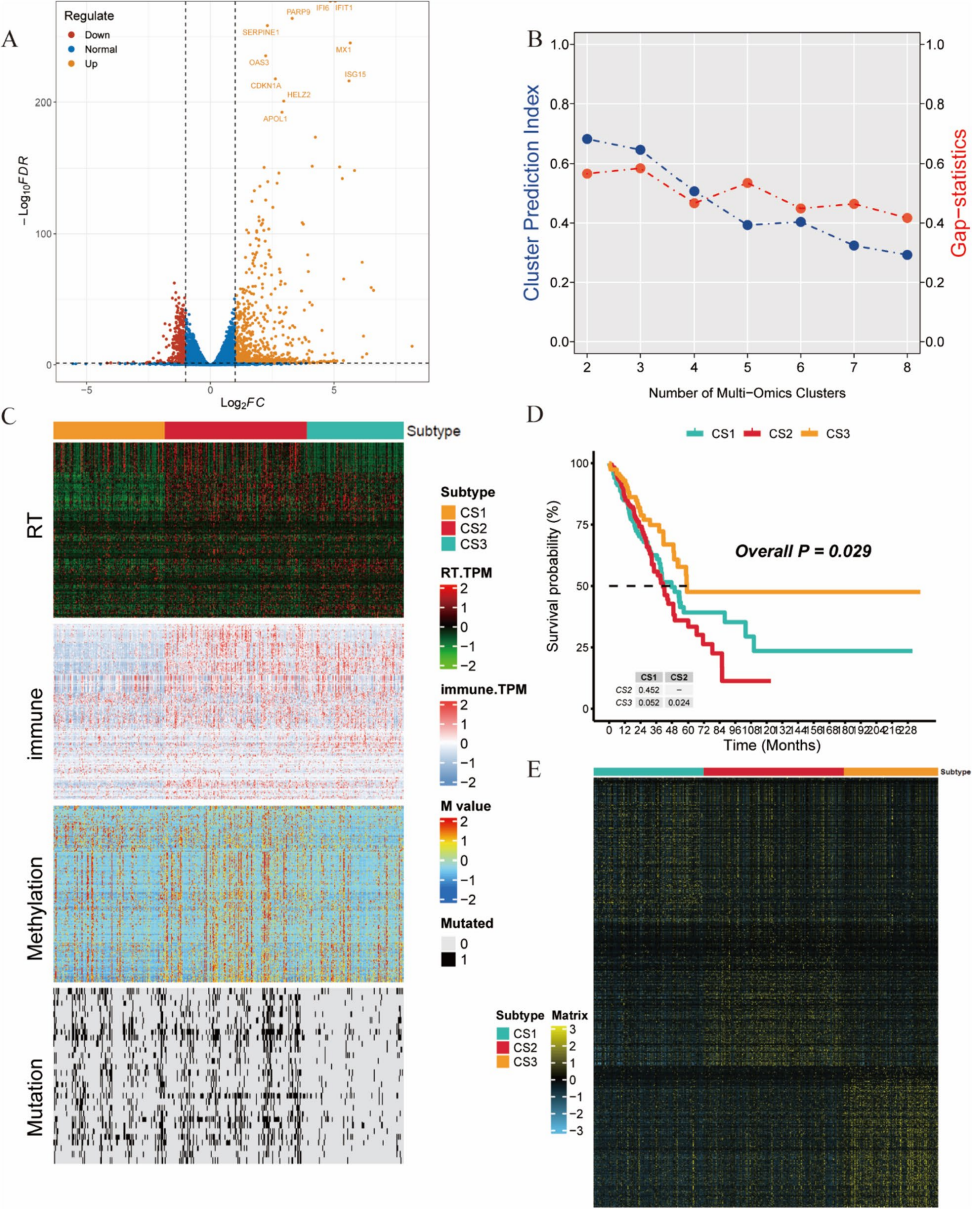
Achieving lung adenocarcinoma classification based on multi-omics data. **A** Volcano plot of differentially expressed genes in A549 cells before and after radiation. **B** Calculation of the CPI and gap statistic to identify the optimal clustering number for LUAD. **C** Using multi-omics data, a comprehensive heatmap displaying the detailed molecular landscapes of radio-related genes, immune-related genes, DNA methylation, and gene mutations in the three subtypes was constructed. **D** Kaplan‒Meier survival analysis of OS rates for the three subtypes. **E** Heatmap of upregulated biomarkers in the subgroups

A differential analysis was conducted on the sequencing data of A549 cells before and after radiation, which revealed 1,068 differentially expressed genes, 284 of which exhibited decreased expression, and 784 of which exhibited increased expression after radiation (Fig. 1A). After matching the radiation-related gene, immune-related gene, methylation, and mutation data, 446 TCGA-LUAD samples were included in the subsequent analysis. The number of clusters was estimated using the cluster prediction index (CPI) and gap statistics (Fig. 1B). We conducted the analysis with *k* = 3. Subsequently, we applied 10 algorithms to cluster LUAD patients. The results showed that the SNF algorithm could successfully separate and distinguish LUAD patients when *k* = 3 (Fig. 1C, Additional file 1: Fig. S1). Although the ConsensusClustering classification was meaningful in the TCGA-LUAD dataset, it failed to yield significant results in four independent validation sets. Based on the SNF algorithm, LUAD patients were classified into three subtypes: CS1, CS2, and CS3 (Fig. 1C, Table 1). Survival analysis revealed significant differences in overall survival rates among these three subtypes (*P* = 0.029; Fig. 1D). CS3 patients had a relatively favorable prognosis, while CS2 patients had the worst prognosis. The heatmaps of the upregulated biomarkers in these subgroups are shown in Fig. 1E.

**Table 1:** Baseline characteristics of participants in CS1, CS2, and CS3 LUAD groups

	Comptab.level	Comptab.CS1	Comptab.CS2	Comptab.CS3	Comptab.p	Comptab.test
n		142	182	122		
Age (%)	< = 65	78 (54.9)	82 (45.1)	52 (42.6)	0.311	
	> 65	59 (41.5)	92 (50.5)	64 (52.5)		
	Unknown	5 ( 3.5)	8 ( 4.4)	6 ( 4.9)		
M (%)	M0	95 (66.9)	121 (66.5)	71 (58.2)	0.134	
	M1	9 ( 6.3)	7 ( 3.8)	3 ( 2.5)		
	Unknown	38 (26.8)	54 (29.7)	48 (39.3)		
N (%)	N0	92 (64.8)	114 (62.6)	84 (68.9)	0.076	Exact
	N1	21 (14.8)	39 (21.4)	21 (17.2)		
	N2	27 (19.0)	26 (14.3)	11 ( 9.0)		
	N3	0 ( 0.0)	1 ( 0.5)	0 ( 0.0)		
	Unknown	2 ( 1.4)	2 ( 1.1)	6 ( 4.9)		
T (%)	T1	38 (26.8)	59 (32.4)	54 (44.3)	0.012	Exact
	T2	87 (61.3)	100 (54.9)	51 (41.8)		
	T3	13 ( 9.2)	14 ( 7.7)	11 ( 9.0)		
	T4	4 ( 2.8)	9 ( 4.9)	3 ( 2.5)		
	Unknown	0 ( 0.0)	0 ( 0.0)	3 ( 2.5)		
Gender (%)	Female	58 (40.8)	100 (54.9)	82 (67.2)	< 0.001	
	Male	84 (59.2)	82 (45.1)	40 (32.8)		
Stage (%)	Stage 1	76 (53.5)	91 (50.0)	76 (62.3)	0.312	Exact
	Stage 2	31 (21.8)	49 (26.9)	27 (22.1)		
	Stage 3	26 (18.3)	32 (17.6)	14 (11.5)		
	Stage 4	9 ( 6.3)	7 ( 3.8)	4 ( 3.3)		
	Unknown	0 ( 0.0)	3 ( 1.6)	1 ( 0.8)		

### Evaluation of genetic alteration for three subtypes

**Fig. 2:**
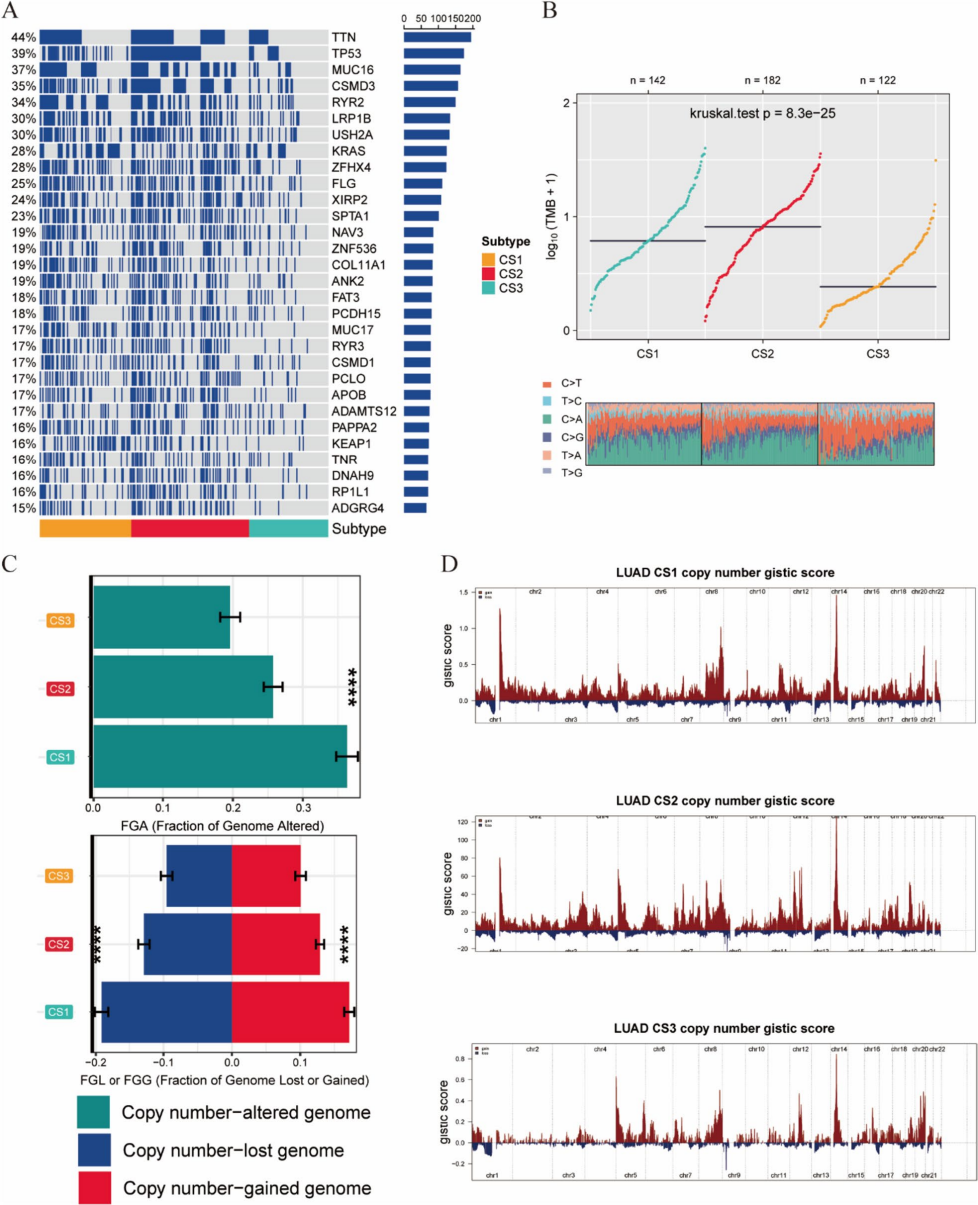
Changes at the gene level in each subtype. **A** Waterfall plot showing significantly mutated genes in each subtype. **B** Analysis of TMB between subgroups. **C** Distribution of FGA and FGG/FGL. The bar chart represents the mean ± SD. **D** Copy number amplifications and deletions of 22 chromosomes in the three subgroups

Gene mutations and CNVs play critical roles in the initiation and progression of tumors [30]. Therefore, we compared the gene alterations among the different subtypes. First, we compared the differences in the mutations of individual genes among these three subtypes. TTN, TP53, MUC16, CSMD3, and RYR2 were the five genes with the highest mutation frequencies in LUAD patients. Compared to those in the CS3 group, the CS1 and CS2 groups had higher mutation frequencies (Fig. 2A). The mutation frequencies of the TTN and TP53 genes were slightly higher in the CS2 group than in the CS1 group, while the KRAS gene had the highest mutation frequency in the CS1 group (Table 2). The TMB is a promising and clinically validated biomarker for immune checkpoint inhibitors (ICIs) [31]. The TMB was highest in the CS2 group, while it was lowest in the CS3 group (Fig. 2B). High TMB represents the presence of new antigens in tumor cells, increasing the likelihood of recognition by the immune system and response to immunotherapy [32, 33]. Therefore, patients in the CS2 group may have a better response to immunotherapy. We also evaluated CNVs in the three subtypes by calculating the FGA score to study chromosomal instability. We found that, compared with the other subtypes, CS3 had better chromosomal stability and significantly lower copy number loss or gain. To explore the differences in somatic copy number alterations (SCNAs) among the different subtypes, we analyzed the changes in chromosomal regions using GISTIC 2.0. We plotted copy number amplifications and deletions based on G-scores. We found significant copy number amplifications on chromosomes 2 and 8 in CS1 patients and on chromosomes 3, 7, and 12 in CS2 patients (Fig. 2D). These results revealed the reasons for the good prognosis of CS3 and the poor prognosis of CS1 and CS2 at the gene mutation and copy number levels.

**Table 2:** Independent test of subtypes and mutations

Gene (Mutated)	TMB	CS1	CS2	CS3	*p* value	*p* adj
TP53	174 (39%)	44 (31.0%)	107 (58.8%)	23 (18.9%)	8.84e-13	2.65e-11
TTN	195 (44%)	64 (45.1%)	102 (56.0%)	29 (23.8%)	1.08e-07	4.63e-07
MUC16	164 (37%)	64 (45.1%)	83 (45.6%)	17 (13.9%)	1.05e-09	7.88e-09
CSMD3	157 (35%)	46 (32.4%)	94 (51.6%)	17 (13.9%)	2.65e-11	3.97e-10
RYR2	150 (34%)	63 (44.4%)	71 (39.0%)	16 (13.1%)	1.62e-08	9.72e-08
LRP1B	134 (30%)	52 (36.6%)	68 (37.4%)	14 (11.5%)	2.15e-07	8.06e-07
USH2A	132 (30%)	49 (34.5%)	67 (36.8%)	16 (13.1%)	5.66e-06	9.99e-06
ZFHX4	123 (28%)	49 (34.5%)	61 (33.5%)	13 (10.7%)	1.35e-06	3.12e-06
KRAS	124 (28%)	62 (43.7%)	39 (21.4%)	23 (18.9%)	3.18e-06	6.36e-06
XIRP2	108 (24%)	35 (24.6%)	60 (33.0%)	13 (10.7%)	2.39e-05	3.77e-05
FLG	111 (25%)	43 (30.3%)	55 (30.2%)	13 (10.7%)	4.32e-05	6.17e-05
SPTA1	101 (23%)	40 (28.2%)	53 (29.1%)	8 (6.6%)	4.84e-07	1.45e-06
FAT3	80 (18%)	30 (21.1%)	41 (22.5%)	9 (7.4%)	7.88e-04	9.09e-04
NAV3	85 (19%)	34 (23.9%)	42 (23.1%)	9 (7.4%)	2.21e-04	2.88e-04
COL11A1	83 (19%)	40 (28.2%)	37 (20.3%)	6 (4.9%)	1.02e-06	2.78e-06
ZNF536	85 (19%)	27 (19.0%)	49 (26.9%)	9 (7.4%)	5.55e-05	7.57e-05
CSMD1	77 (17%)	34 (23.9%)	32 (17.6%)	11 (9.0%)	4.89e-03	5.24e-03
ANK2	83 (19%)	34 (23.9%)	40 (22.0%)	9 (7.4%)	3.40e-04	4.25e-04
PCLO	77 (17%)	26 (18.3%)	45 (24.7%)	6 (4.9%)	9.62e-06	1.60e-05
PCDH15	80 (18%)	29 (20.4%)	41 (22.5%)	10 (8.2%)	2.35e-03	2.61e-03
MUC17	78 (18%)	36 (25.4%)	39 (21.4%)	3 (2.5%)	3.44e-08	1.72e-07
RYR3	78 (18%)	33 (23.2%)	41 (22.5%)	4 (3.3%)	4.31e-07	1.44e-06
APOB	77 (17%)	24 (16.9%)	47 (25.8%)	6 (4.9%)	3.73e-06	6.99e-06
ADAMTS12	74 (17%)	20 (14.1%)	44 (24.2%)	10 (8.2%)	6.88e-04	8.26e-04
KEAP1	72 (16%)	43 (30.3%)	26 (14.3%)	3 (2.5%)	7.29e-10	7.29e-09
TNR	70 (16%)	30 (21.1%)	30 (16.5%)	10 (8.2%)	1.18e-02	1.22e-02
PAPPA2	73 (16%)	27 (19.0%)	35 (19.2%)	11 (9.0%)	2.77e-02	2.77e-02
DNAH9	70 (16%)	23 (16.2%)	43 (23.6%)	4 (3.3%)	1.50e-06	3.21e-06
RP1L1	70 (16%)	21 (14.8%)	43 (23.6%)	6 (4.9%)	2.60e-05	3.90e-05
ADGRG4	65 (15%)	22 (15.5%)	40 (22.0%)	3 (2.5%)	1.18e-06	2.95e-06

### Evaluation of the immune microenvironment and response to immunotherapy and chemotherapy

**Fig. 3:**
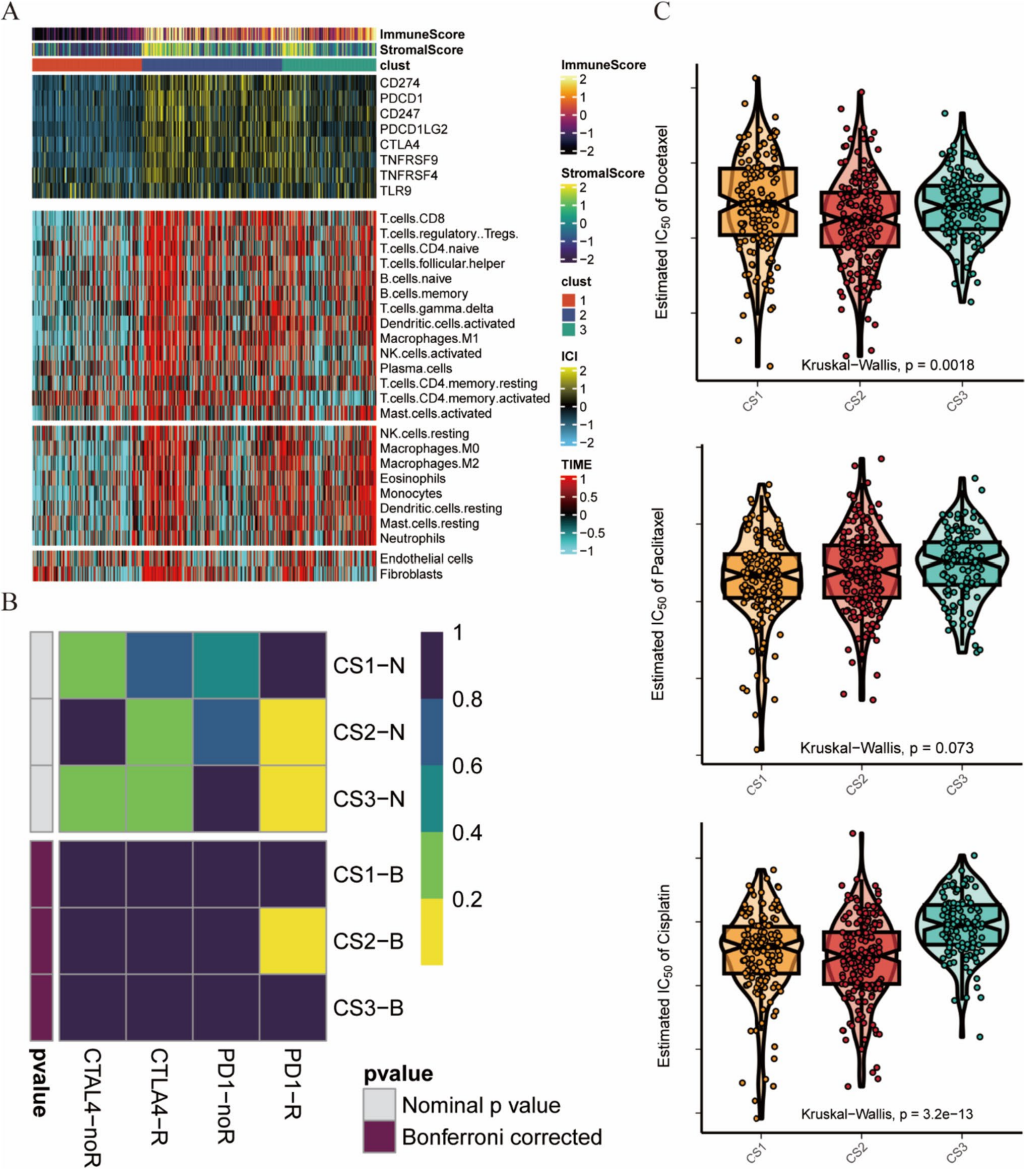
Differences in the immune microenvironment and sensitivity to immunotherapy and chemotherapy among subtypes. **A** Heatmap showing the expression of immune checkpoint genes and the levels of tumor-infiltrating lymphocytes in each subtype. **B** Submap analysis displaying patient responses to PD-1 immunotherapy in each subtype. **C** IC50 values of commonly used chemotherapy drugs

Considering the crucial role of the immune system in the progression of LUAD, we investigated the abundance of immune cell distributions and immune checkpoint expression in these three subtypes. Patients in the CS2 group exhibited the highest levels of immune checkpoint expression and immune cell infiltration, while those in the CS1 group had the lowest levels (Fig. 3A). Subsequently, we investigated the treatment response of these three subtypes to ICIs. We used a subtype mapping approach to predict the clinical response to immune checkpoint blockade. The CS2 patients were more likely to benefit from anti-PD-1 immunotherapy (*p* < 0.05, Bonferroni corrected), consistent with previous findings on TMB (Fig. 3B). We also analyzed the drug sensitivity of these three subtypes to cisplatin, paclitaxel, and docetaxel. The patients in the CS2 group were most sensitive to docetaxel and cisplatin, while those in the CS1 group exhibited the highest sensitivity to paclitaxel (Fig. 3C).

### Functional enrichment analysis and single-cell analysis of subtypes

**Fig. 4:**
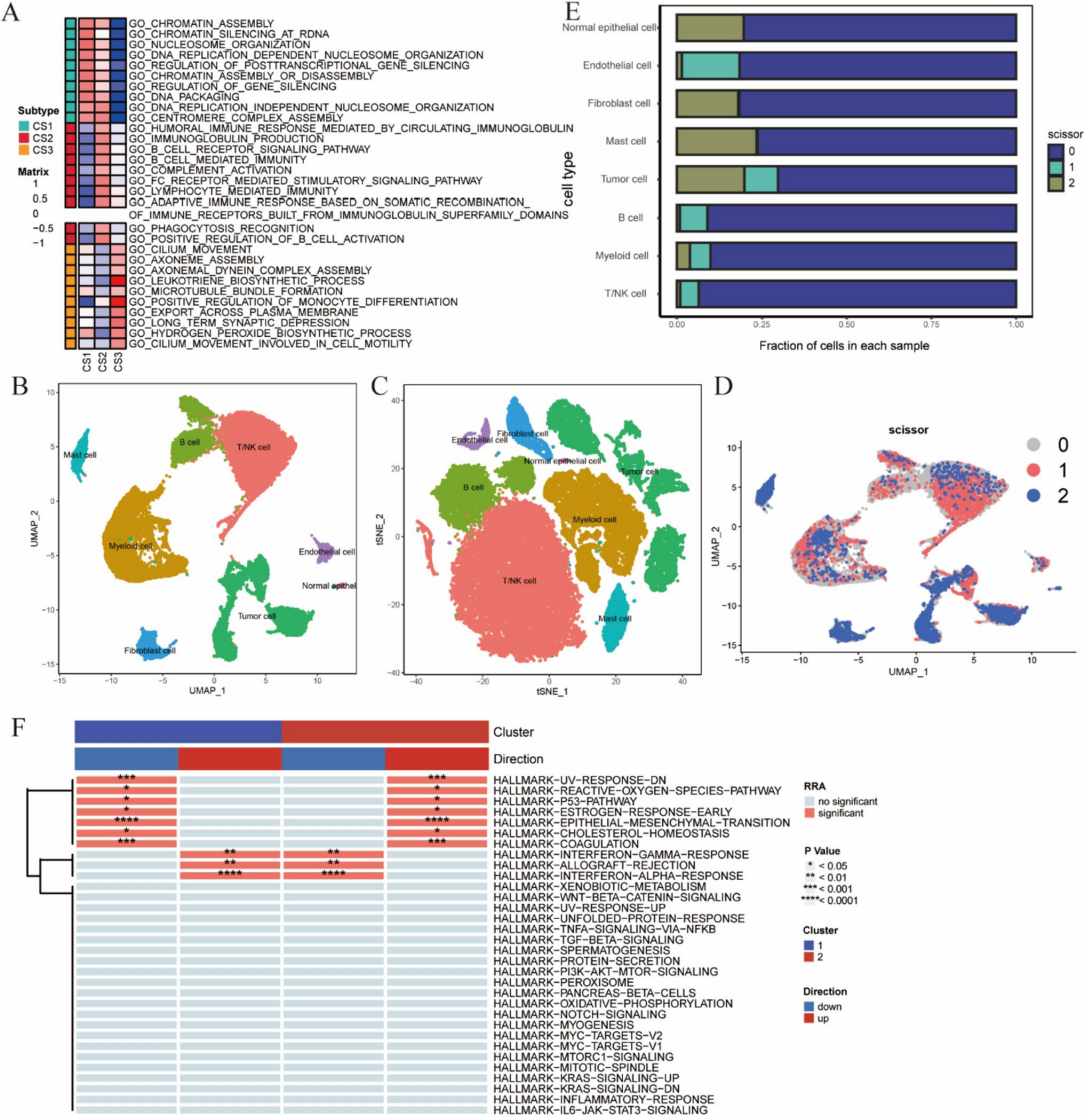
Integration analysis of single-cell and bulk sequencing data. **A** GO enrichment analysis displaying upregulated and downregulated pathways in each subtype. **B** UMAP dimensionality reduction clustering plot. **C** tSNE dimensionality reduction clustering plot. **D** UMAP plot of scissor cell distribution. **E** Proportions of specific cell types predicted by the scissor algorithm in each cell cluster. **F** Heatmap of single-cell functional enrichment analysis

We conducted functional enrichment analysis of the three subtypes using the GSEA algorithm (Fig. 4A). The results indicate that the CS1 group activates biological processes related to chromatin assembly or disassembly, chromatin silencing, and DNA packaging, which are associated with the cell nucleus. The CS2 group also exhibited activation tendencies in these biological processes, but the CS3 group exhibited inhibition of these life activities. Moreover, CS2 is primarily associated with the production of immunoglobulins, B-cell-mediated immunity, lymphocyte-mediated immunity, and other immune response processes. These biological processes in the CS1 group were inhibited, which was consistent with the results of immune infiltration and may be the reason for the poor immune treatment response in CS1 patients.

We combined single-cell data with bulk data using the scissor algorithm to further explore the differences in tumor immune microenvironment subtypes. Cell annotation was performed after gene filtering, normalization, and principal component analysis were performed to obtain eight specific cell types (Fig. 4B, C). Subsequently, using the scissor algorithm, we successfully predicted three cell clusters, labeled 0, 1, and 2. Here, 1 represents scissor-positive cells, 2 denotes scissor-negative cells, and 0 corresponds to cell clusters unrelated to the classification (Fig. 4D). The bar chart shows the proportions of specific cell types predicted by the scissor algorithm in each cluster (Fig. 4E). Cluster C1 was enriched with B cells, T/NK cells, myeloid cells, and endothelial cells. Cluster C2 was enriched with normal epithelial cells, fibroblasts, adipocytes, and tumor cells. Functional enrichment analysis of single-cell data was performed using the UCell, singscore, and ssGSEA algorithms, followed by integration of the results using the RRA algorithm (Fig. 4F). As shown in Fig. 4F, in the scissor + cells, genes related to the IFN-γ response, IFN-α response activation, P53 pathway, and UV response were inhibited, while the opposite was observed in the scissor- cells.

**Fig. 5:**
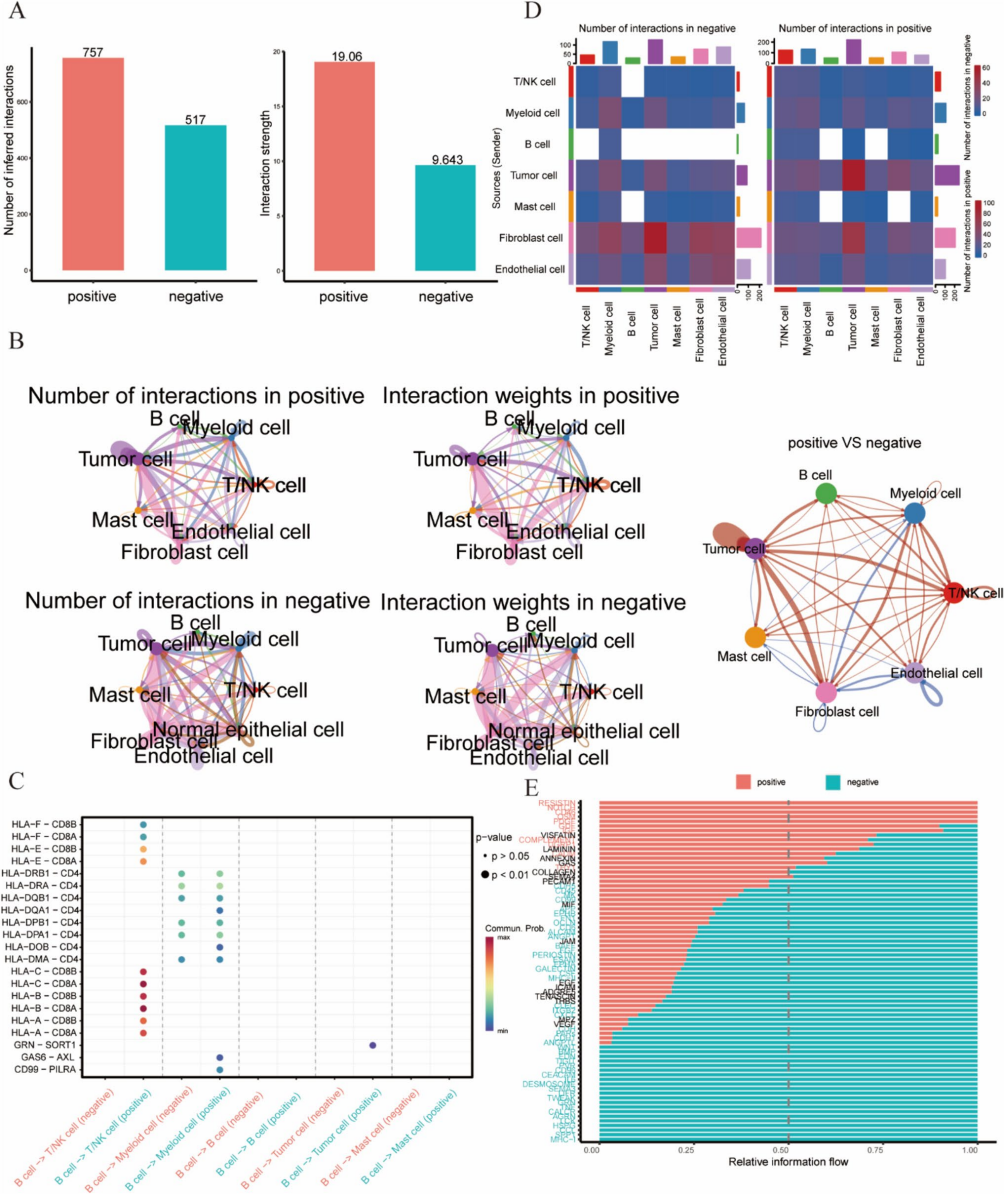
Cell-to-cell communication analysis. **A** Bar graph displaying the strength and quantity of intercellular communication between the scissor + and Scissor- cells. **B** Circular plot of cell-to-cell communication among the major scissor + and scissor- cell types. **C** Ligand–receptor pairs between cells. **D** Heatmap of intercellular communication between the scissor + and scissor- cells. **E** Relative information flow between the scissor + and scissor- cells

We also analyzed the differences in cell communication between scissor + and Scissor- cells, and the scissor + cells had both a higher number and stronger intensity of communication than the scissor- cells (Fig. 5A). Network visualization was generated to display the interactions between different cells (Fig. 5B). Ligand–receptor pair comparisons between cells revealed that the differences in ligand–receptor pairs between the scissor + and scissor- cells were mainly observed for B cells and T/NK cells, while myeloid cells and tumor cells among the scissor + cells exhibited greater communication intensity and significance, which is consistent with the results obtained from the GSEA of these subtypes (Fig. 5C–E). These results suggested that B cells play important roles in the CS2 subtype.

### Validating the reliability of subtypes with four external datasets

**Fig. 6:**
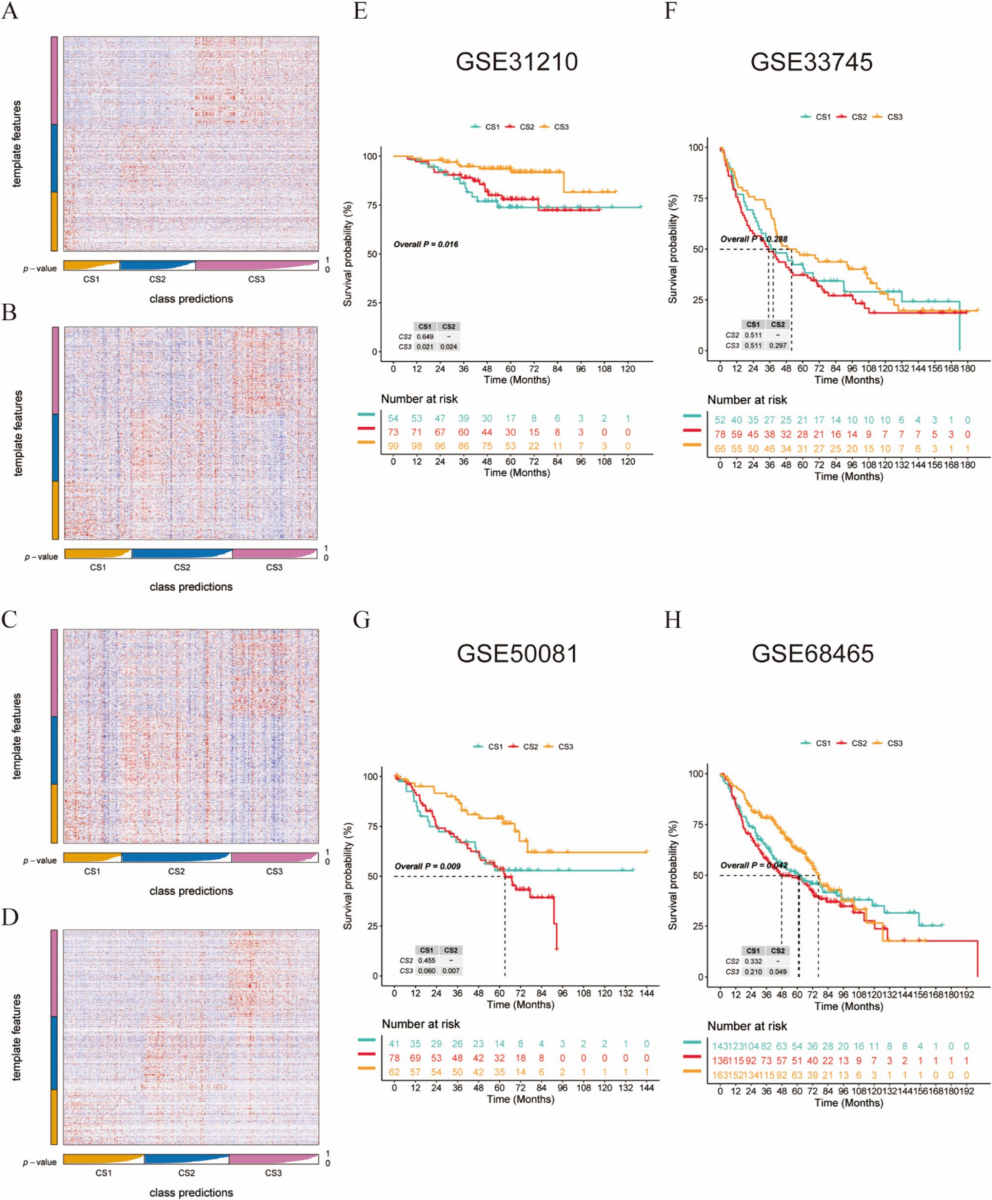
External dataset validation. **A**–**D** Heatmaps of NTP in four external datasets (GSE31210, GSE33745, GSE50081, and GSE68465) generated from subtype-specific upregulated biomarkers identified in the LUAD cohort. **E**–**H** Kaplan‒Meier survival curves predicting the three subtypes in four external datasets

We determined the top 200 upregulated biomarkers (*P* < 0.05) for the three subtypes using 'DESeq2'. Subsequently, we selected four external lung cancer datasets: GSE31210, GSE33745, GSE50081, and GSE68465. These external datasets were used to validate the reliability of the subtypes. Based on the specific upregulation profile of biomarkers in each subtype, the NTP method was used to predict the prognosis for each dataset (Fig. 6A–D). The NTP results, as shown in the figure, demonstrated that CS3 had the best prognosis of all the external validation datasets, while CS1 and CS2 had poorer prognoses, consistent with the original subtype predictions (Fig. 6E–H). Moreover, there was no significant difference in prognosis between CS1 and CS2 patients; however, in some datasets, such as in GSE33745 and GSE68465, CS2 patients had a slightly worse prognosis than CS1 patients. These results indicated the reliability of our subtyping approach.

### Acquisition and validation of marker genes for the CS2 subtype

**Fig. 7:**
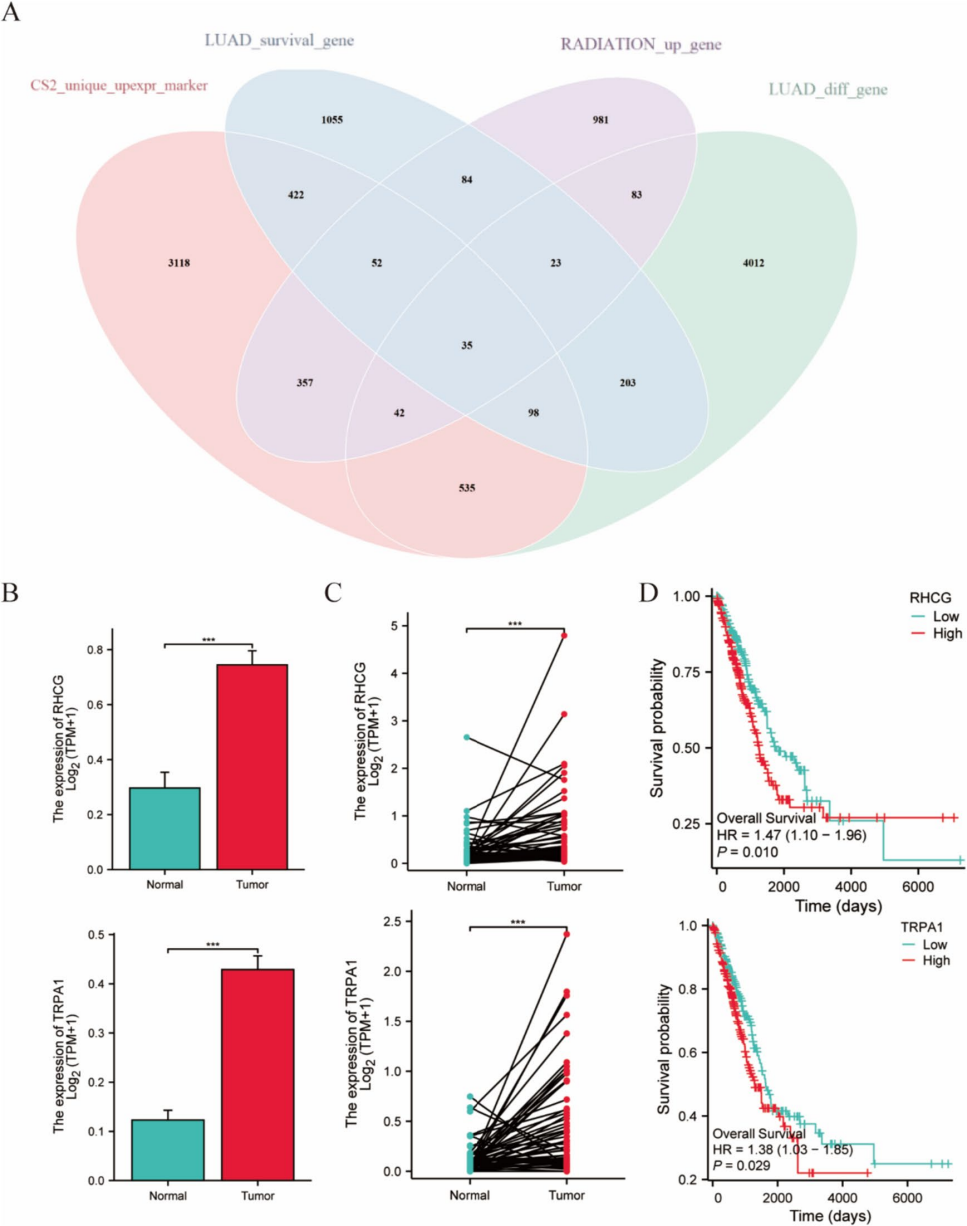
Obtaining CS2 subtype signature genes. **A** Venn diagram showing the intersection of the CS2 signature gene, differential gene in LUAD, differential gene in radiotherapy, and prognostic gene sets. **B** Differences in the expression of TRPA1 and RHCG between tumor tissue and adjacent tissue in LUAD. **C** Differences in TRPA1 and RHCG expression between paired sample tissues from LUAD patients. **D** Kaplan‒Meier curves demonstrating prognostic differences between the high- and low-expression groups of RHCG and TRPA1 in LUAD

We used the edgeR algorithm to perform differential expression analysis between CS2 and the other subtypes. We selected CS2-specific highly expressed marker genes and intersected these genes with differential genes in LUAD, prognostic-related genes, and radiation-enhanced genes, resulting in 35 common genes (Fig. 7A). These genes may play essential roles in the progression and radioresistance of LUAD. We selected the top-ranking gene RHCG and the intermediate-ranking gene TRPA1 for bioinformatics and experimental validation. RHCG and TRPA1 were highly expressed in LUAD tissues and exhibited significant differences in paired samples (Fig. 7B, C). Patients with high RHCG and TRPA1 expression had poor prognoses (Fig. 7D).

### Validation of the impact of RHCG and TRPA1 on radiosensitivity

**Fig. 8:**
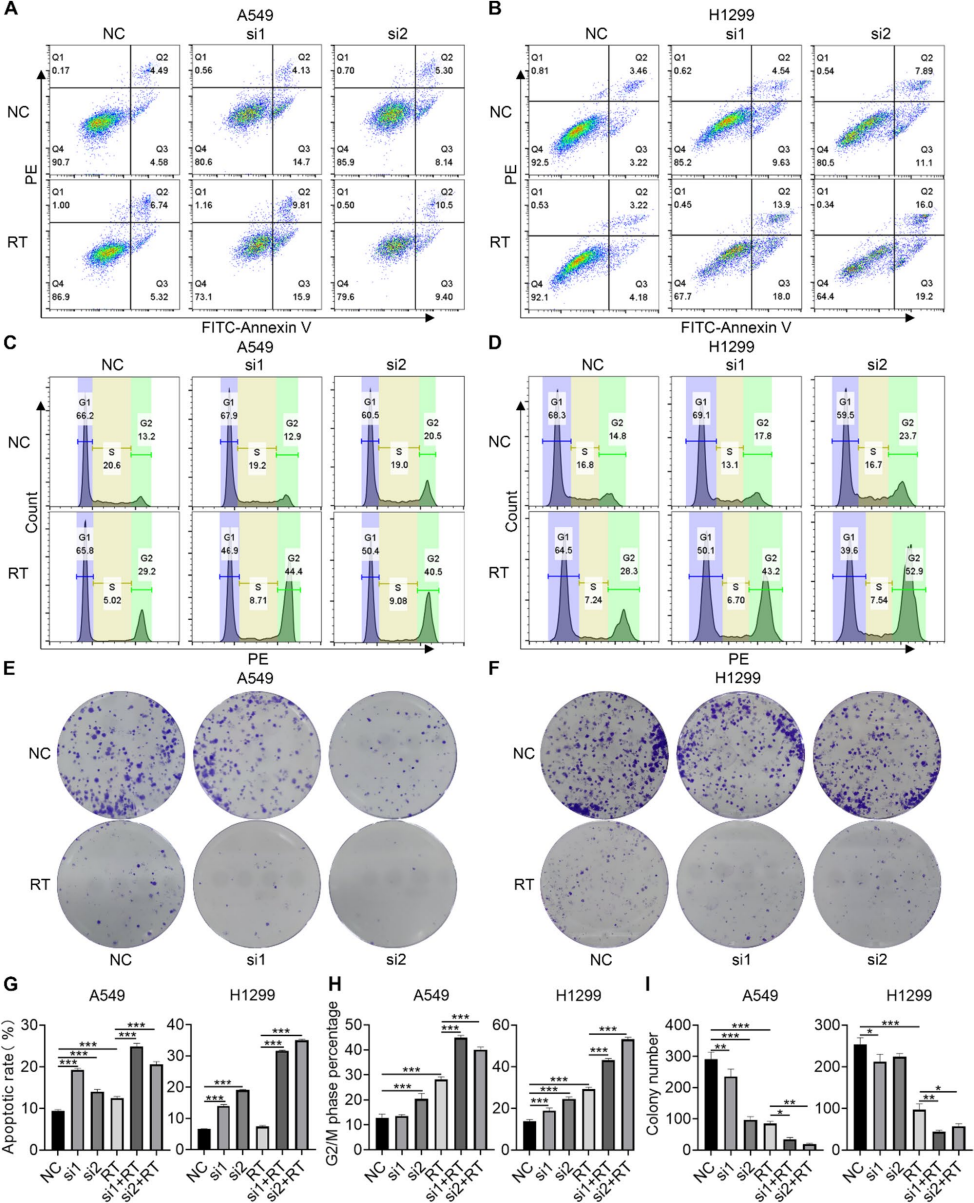
Experiment to verify the effect of TRPA1 expression on the radiosensitivity of LUAD cells. **A-B** Flow cytometry was used to measure the apoptosis rate of A549 and H1299 cells. **C-D** Flow cytometry was used to detect the cell cycle distribution of A549 and H1299 cells. **E–F** Clonogenic assay to test the proliferation of A549 and H1299 cells. **G-I** Quantitative statistical graphs of the above experiments

Increased apoptosis can be induced by radiation. We tested the impact of silencing TRPA1 and the combination of silencing TRPA1 with radiation on apoptosis. TRPA1 silencing increased A549 and H1299 cell apoptosis and radiation-induced apoptosis in A549 cells but not in H1299 cells, considering that H1299 cells were radioresistant. When siTRPA1 was combined with radiation, the rate of apoptosis was more pronounced in both A549 and H1299 cells (Fig. 8A, B). Radiation increased the proportion of G2/M phase cells, and the combination of radiation and TRPA1 silencing significantly increased the proportion of G2/M phase cells among the A549 and H1299 cells compared to that in the radiation group (Fig. 8C, D). To investigate the impact of the combination of radiation and TRPA1 silencing on cell proliferation, colony formation and CCK-8 assays were also conducted. Radiation significantly decreased the number of cell colonies formed by A549 and H1299 cells, and cell colony formation was significantly reduced in the combination group compared to that in the radiation group (Fig. 8E, F). The CCK-8 assay results also showed that the combination treatment significantly reduced the viability of both cell lines (Additional file 1: Fig. S3 A, B). Quantitative statistical analysis of the above experimental results revealed significant differences between the groups (Fig. 8G, H, I).

We also investigated the synergistic antitumor effect of RHCG silencing and radiation on A549 and PC9 cells (Additional file 1: Fig. S2 C, D). Flow cytometry was used to examine the impact of radiation and RHCG silencing on apoptosis. RHCG knockdown, in combination with radiation, enhanced cell apoptosis (Fig. S4A, B). Radiation caused an increase in the number of G2/M phase cells, while the combination of RHCG silencing and radiation led to an even greater increase (Fig. S4 C, D). Colony formation and CCK-8 assays demonstrated that RHCG silencing enhanced the radiation-induced decrease in cell viability (Fig. S4 E, F; Additional file 1: Fig. S3 C, D). Quantitative statistical analysis of the above experimental results revealed significant differences between the groups (Fig. S4G, H, I).

## Discussion

In this study, we successfully established a subtype classifier using the SNF algorithm, which is associated with radiation, immune responses, DNA methylation, and somatic mutations. TCGA-LUAD patients were classified into three subtypes. Among these three subtypes, patients in the CS3 group had a better prognosis than patients in the CS1 and CS2 groups. Consistent results were obtained in four independent external datasets.

Regarding genetic alterations, the CS1 and CS2 groups had higher gene mutation frequencies than the CS3 group, which may be the reason for the poorer prognosis of the CS1 and CS2 groups. The CS2 group had higher TTN and TP53 gene mutation frequencies than the CS1 group, while the KRAS gene mutation frequency was highest in the CS1 group. In the early stages of carcinogenesis, KRAS mutation promotes the survival, invasion, and migration of cancer cells [34]. In LUAD patients, the presence of TP53 mutations is associated with shorter overall survival [35]. TTN gene mutations are related to myofilament dysfunction and abnormal muscle fiber growth. In recent years, the relationship between TTN gene mutations and solid tumors has received widespread attention. LUAD patients with TTN mutations have an inflammatory tumor microenvironment and high levels of activated immune cells. TTN mutation may be a potential predictive biomarker for LUAD patients receiving ICI therapy [36]. The TMB was higher in the CS2 group than in the other group, while the CS3 group had a lower TMB than the other groups. Patients with high TMB are more likely to be recognized by the immune system and to respond to immunotherapy. Therefore, the CS2 group may respond better to immune therapy, consistent with the previous immunotherapy response analysis results.

Immunotherapy provides a promising and innovative approach for the treatment of cancer. By harnessing the power of the immune system, immunotherapy has shown significant efficacy in treating various types of cancer, improving outcomes, and offering new hope to patients worldwide [37, 38]. However, despite the tremendous success of immunotherapy, some limiting factors, such as tumor heterogeneity, primary and acquired resistance, side effects, and toxicity, still hinder its widespread application [39]. Therefore, utilizing specific biomarkers to distinguish between sensitive and insensitive populations is crucial. In the era of precision cancer treatment, our established LUAD classification system holds a great potential for predicting and evaluating the effects of immunotherapy on LUAD patients. We utilized the SubMap method to have a higher likelihood of obtaining a better response to PD-1 immunotherapy in CS2 patients. Furthermore, considering that chemotherapy is a standard lung cancer treatment, we estimated the chemosensitivity of each sample based on the IC50 value. The results showed that patients in the CS2 group were most sensitive to docetaxel and cisplatin, while patients in the CS1 group were most sensitive to paclitaxel.

The tumor microenvironment is crucial for tumor initiation, progression, and immunity [40]. Studies have shown that tumor-infiltrating B cells are essential regulators of lung cancer progression [41]. Under tumor microenvironment signaling, B cells infiltrate, proliferate, and develop within tumors. Tumor-infiltrating B cells exert antitumor immune responses by secreting tumor-specific antibodies, promoting T-cell responses, and maintaining the structure and function of tertiary lymphoid structures, all of which are associated with favorable outcomes in lung cancer patients. However, as multifaceted effectors, B cells can also develop an immunosuppressive phenotype characterized by the secretion of IL-10, leading to tumor progression [42]. We used the scissor algorithm combined with molecular typing and single-cell data to uncover the underlying mechanisms of the subtypes. The results showed that B cells play an essential role in CS2 subtyping, which is consistent with the functional enrichment results, suggesting that B cells, as potential targets, play a crucial role in immunotherapy.

The Rh family C glycoprotein (RHCG) is an electroneutral and bidirectional ammonia transporter that can regulate ammonia secretion across epithelia [43]. Research has shown that RHCG plays an important role in various cancers, such as cervical squamous cell carcinoma and esophageal cancer, but has a procarcinogenic effect on gastric cancer [44]. RHCG affects the proliferation, motility, and metastasis of tumor cells However, no study has explored the expression and potential functions of RHCG in LUAD. Transient receptor potential cation channel subfamily A member 1 (TRPA1) is a nonselective cation channel that plays a vital role in sensation and pain perception [45]. Emerging evidence suggests that TRPA1 may have significant implications for the occurrence and development of cancer. High expression of TRPA1 has been observed in several types of cancer, including breast, lung, pancreatic, and colorectal cancer [46–48]. Studies have shown that activation of TRPA1 can promote cancer cell growth, invasion, and metastasis, which makes TRPA1 an attractive target for therapeutic intervention. Overall, the roles of RHCG and TRPA1 in the context of radiotherapy and immunotherapy in LUAD have not yet been determined. Our findings suggest that the inhibition of RHCG and TRPA1 enhances the sensitivity of lung cancer cells to radiation and may provide a new target for the combination of radiotherapy and immunotherapy for lung cancer treatment. Further research can identify the detailed mechanisms of these molecules in tumor radiation therapy and immunotherapy, providing a theoretical basis for developing new treatment strategies.

However, several limitations in the current study should be considered when interpreting our results. First, transcriptome analysis can only reflect changes at the mRNA level rather than overall changes. Second, our study focused on investigating the reasons for poor prognosis in the CS2 group, but there was insufficient research on the differences between the CS2 and CS1 groups. The CS1 group, which includes cold tumors, may represent a population that is insensitive to immunotherapy in the clinic. Therefore, further exploration of the differences between these two groups may provide a potential solution to address the poor response to immune checkpoint inhibitors. Finally, our results need to be validated using in vivo experiments and clinical samples.

## Conclusion

In conclusion, we successfully classified LUAD into three subtypes by integrating various omics data. These subtypes are closely associated with patient prognosis, tumor microenvironment characteristics, molecular features, chemotherapy, and immunotherapy response. Our findings may contribute to a better understanding and exploration of the heterogeneity of LUAD and its underlying pathological mechanisms. We hope that this innovative classification method for LUAD will further contribute to precision medicine and provide insights for developing rational clinical strategies for radiotherapy and immunotherapy.

### Supplementary Information

Below is the link to the electronic supplementary material.

Supplementary file1 (DOCX 1863 kb)

## Data Availability

The data used to support the findings of this study are available from the corresponding author upon request.

## Abbreviations

CNV: Copy number variation
GO: Gene ontology
GDSC: Genomics of drug sensitivity in cancer
ICI: Immune checkpoint inhibitor
IC50: Half-maximal inhibitory concentration
LUAD: Lung adenocarcinoma
OS: Overall survival
SNF: Similarity network fusion.
TCGA: The cancer genome atlas
TMB: Tumor mutation burden
