# Supplementary material for: Molecular classification reveals the sensitivity of lung adenocarcinoma to radiotherapy and immunotherapy: multi-omics clustering based on similarity network fusion
Source: Cancer Immunol Immunother. 2024 Mar 2;73(4):71. doi: 10.1007/s00262-024-03657-x (PMC10908647; doi:10.1007/s00262-024-03657-x)
Supplement: Supplementary file 1 — Supplementary file1 (DOCX 1863 kb) [file 262_2024_3657_MOESM1_ESM.docx]

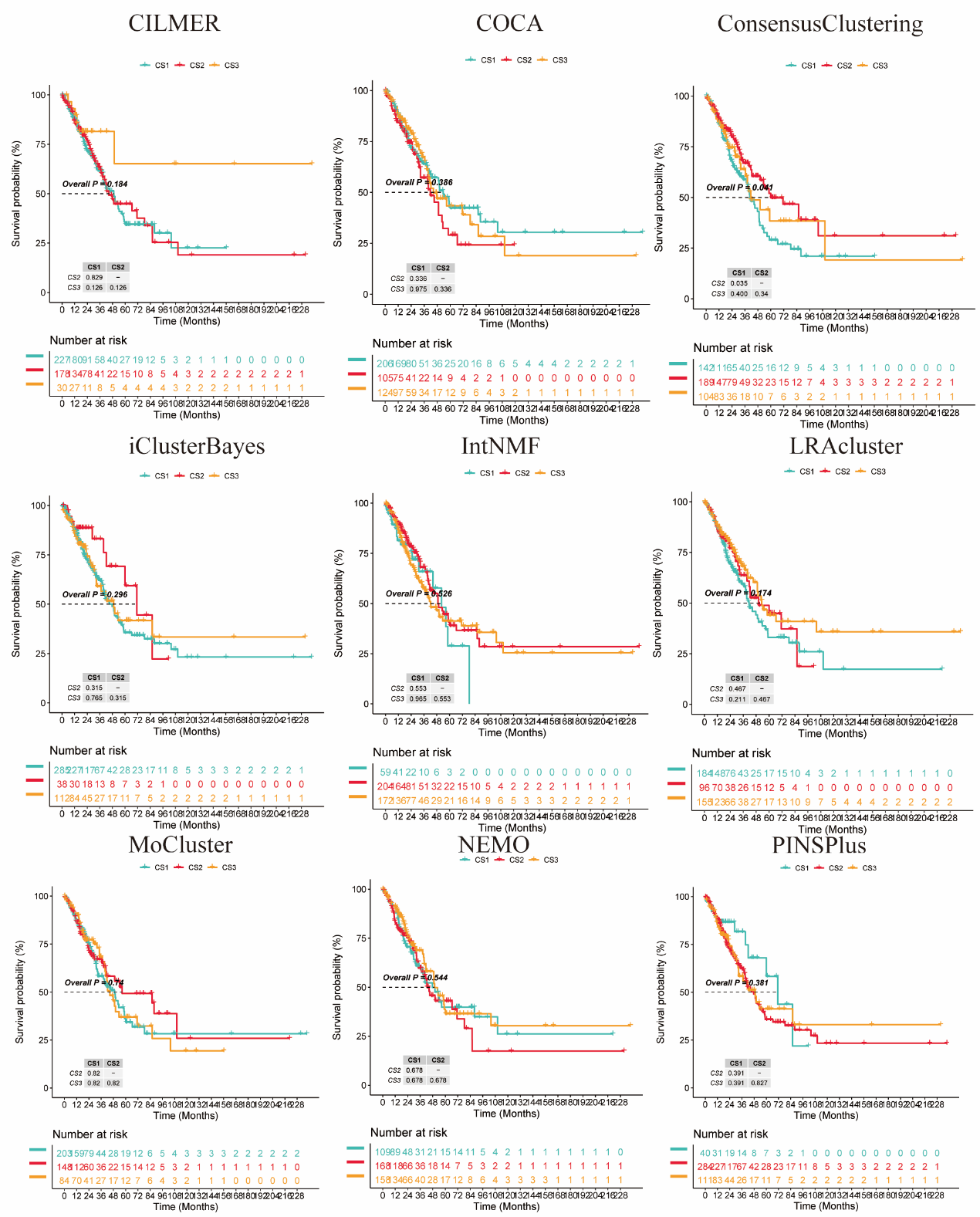


Fig. S1: Kaplan-Meier survival curves of LUAD subtypes based on the iClusterBayes, moCluster, CIMLR, IntNMF, ConsensusClustering, COCA, NEMO, PINSPlus, and LRA classification methods.


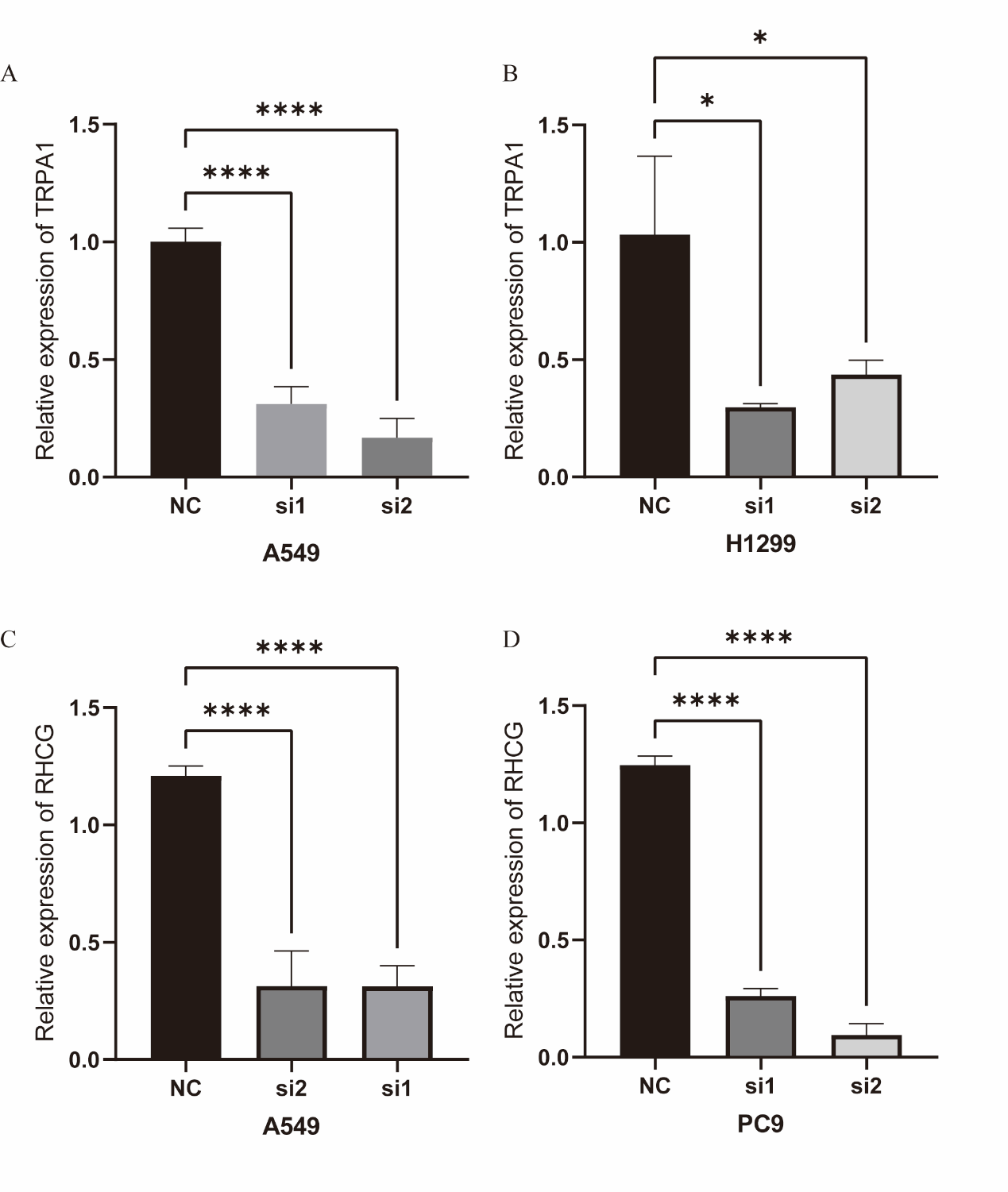


Fig. S2: siRNA knockdown efficiency validation. qRT-PCR analysis of TRPA1 expression in A549 (A) and H1299 (B) cells transfected with TRPA1 siRNA (KD) and normal controls (NC). qRT-PCR analysis of RHCG expression in A549 (C) and PC9 (D) cells transfected with RHCG siRNA (KD) and normal controls (NC)


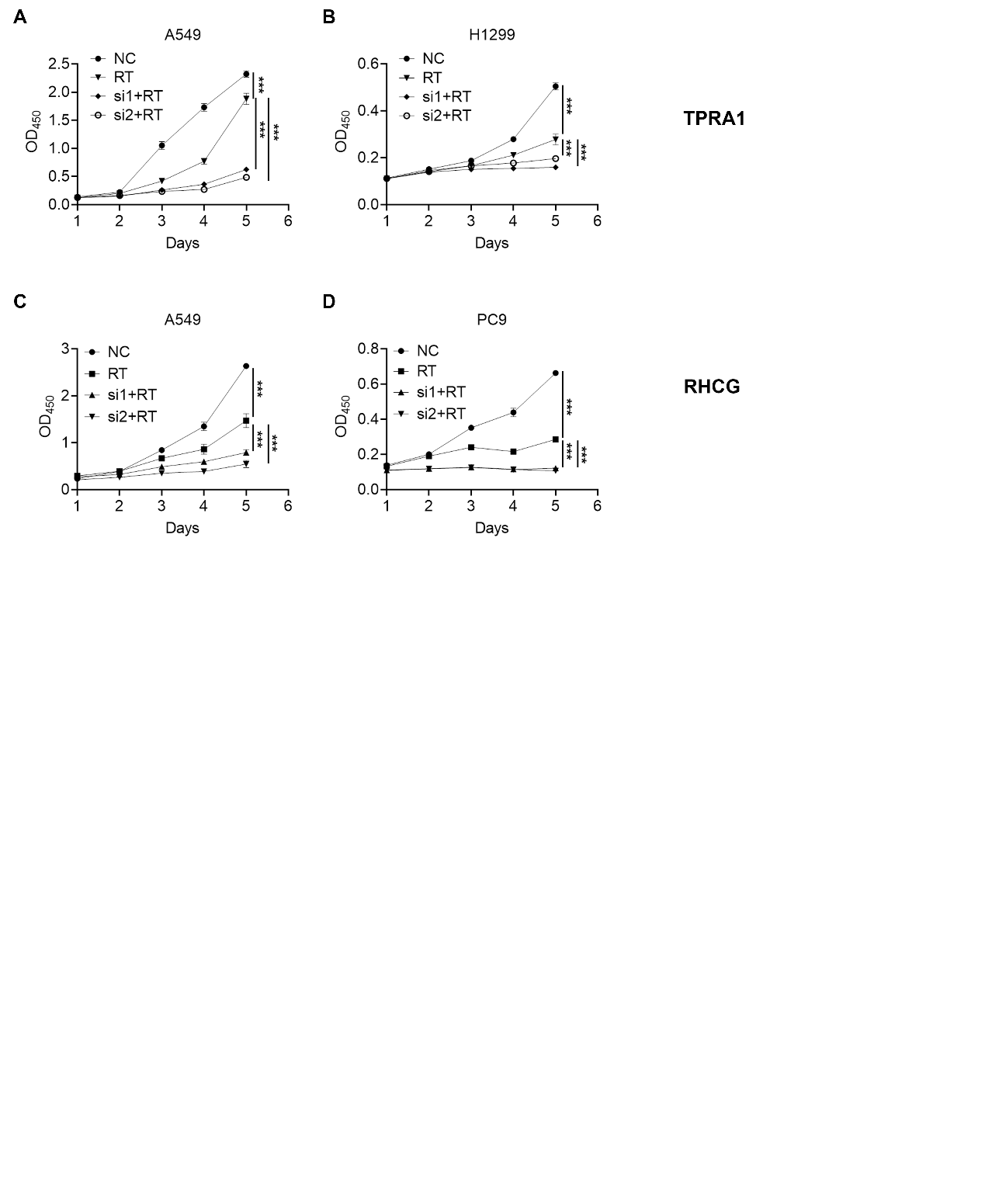


Fig. S3: CCK8 assay was performed to evaluate cell proliferation. CCK8 assay was conducted to assess cell proliferation of A549 (A) and 1299 (B) cells after knockdown of TRPA1 combined with radiotherapy. CCK8 assay was used to determine cell proliferation of A549 (C) and PC9 (D) cells after knockdown of RHCG combined with radiotherapy.


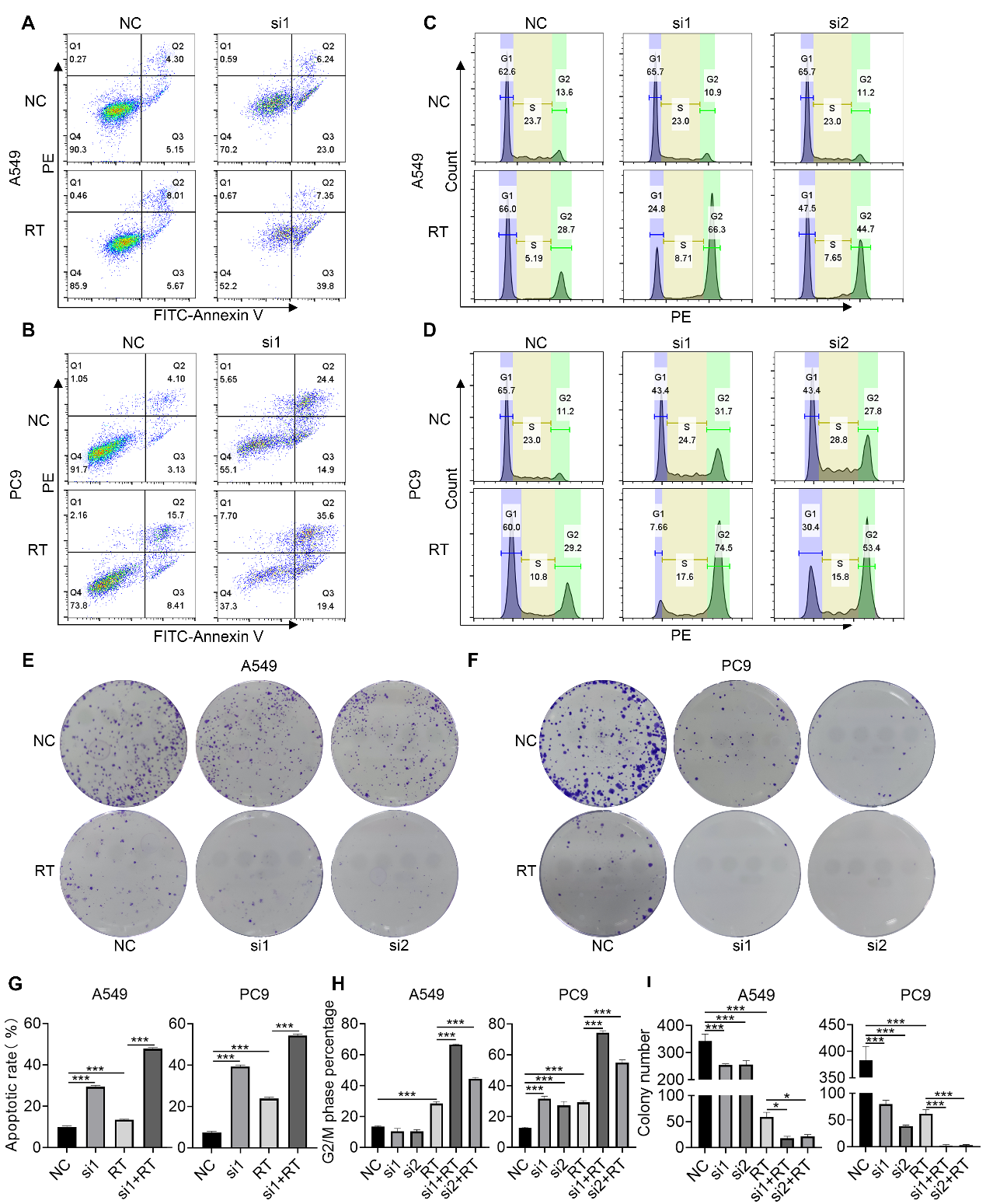


Fig. S4: Experiment to verify the effect of RHCG expression on the radiosensitivity of LUAD cells. A-B, Flow cytometry to detect the apoptosis rate of A549 and PC9 cells. C-D, Flow cytometry to detect the cell cycle of A549 and PC9 cells. E-F, Clonogenic assay to test the proliferation of A549 and PC9 cells. G-I, Quantitative statistical graphs of the above experiments.

Table S1 siRNA sequence

|  | **sense（5'-3'）** | **antisence（5'-3'）** |
| --- | --- | --- |
| **NC** | UUCUCCGAACGUGUCACGU(dT)(dT) | UAUCUGACAGGGCUUGAGC(dT)(dT) |
| **RHCG-si1** | GCGUGGAGAACCUCAUCAA(dT)(dT) | UUGAUGAGGUUCUCCACGC(dT)(dT) |
| **RHCG-si2** | GCUUUGAGGAUGCGGUCUA(dT)(dT) | UAGACCGCAUCCUCAAAGC(dT)(dT) |
| **TRPA1-si1** | GCAUUUAACUGUACAGCAA(dT)(dT) | UUGCUGUACAGUUAAAUGC(dT)(dT) |
| **TRPA1-si2** | GUAGACAGUUGCACAUUAA(dT)(dT) | UUAAUGUGCAACUGUCUAC(dT)(dT) |

Table S2 Primers used for RT-PCR.

| **Gene** | **Forward Primer (5’-3’)** | **Reverse Primer (5’-3’)** |
| --- | --- | --- |
| RHCG | GGTTCCACTTCTTACAAGACCG | GGGCTGACTTTACCCAGAACT |
| TRPA1 | CCAGGGCGTTGTCTATGAGG | AGGTGTCCATATCGTCACATCT |
| GAPDH | GGAGCGAGATCCCTCCAAAAT | GGCTGTTGTCATACTTCTCATGG |
